# Maternal Urinary Fluoride and Child Neurobehavior at Age 36 Months

**DOI:** 10.1001/jamanetworkopen.2024.11987

**Published:** 2024-05-20

**Authors:** Ashley J. Malin, Sandrah P. Eckel, Howard Hu, E. Angeles Martinez-Mier, Ixel Hernandez-Castro, Tingyu Yang, Shohreh F. Farzan, Rima Habre, Carrie V. Breton, Theresa M. Bastain

**Affiliations:** 1 Department of Epidemiology, College of Public Health and Health Professions University of Florida, Gainesville; 2 College of Medicine, University of Florida, Gainesville; 3 Department of Population and Public Health Sciences, Keck School of Medicine of University of Southern California, Los Angeles; 4 Department of Dental Public Health and Dental Informatics, School of Dentistry, Indiana University, Indianapolis

## Abstract

**Importance:**

Recent studies in Canadian and Mexican populations suggest an association of higher prenatal fluoride exposure with poorer neurobehavioral development, but whether this association holds for US-based populations is unknown.

**Objective:**

To examine associations of third trimester maternal urinary fluoride (MUF) with child neurobehavior at age 3 years in the US.

**Design, Setting, and Participants:**

This prospective cohort study utilized urine samples archived from 2017 to 2020 and neurobehavioral data assessed from 2020 to 2023 from the Maternal and Developmental Risks from Environmental and Social Stressors (MADRES) pregnancy cohort, which consisted of predominately Hispanic women residing in Los Angeles, California. Cohort eligibility criteria at recruitment included being 18 years of age or older, less than 30 weeks’ gestation, and a fluent English or Spanish speaker. Exclusion criteria included having a disability preventing participation or provision of informed consent, being HIV positive or incarcerated, and having a multiple gestation pregnancy. There were 263 mother-child pairs who completed the 3-year study visit. In this analysis, women who reported prenatal smoking were excluded. Data analysis was conducted from October 2022 to March 2024.

**Exposure:**

Specific gravity-adjusted MUF (MUF_SG_), a biomarker of prenatal fluoride exposure.

**Main Outcomes and Measures:**

Neurobehavior was quantified using the Preschool Child Behavior Checklist (CBCL), which included composite scores for Total Problems, Internalizing Problems, and Externalizing Problems. CBCL composite T scores range from 28 to 100. T scores from 60 to 63 are in the borderline clinical range, whereas scores above 63 are in the clinical range. Linear and logistic regression models adjusted for covariates were conducted.

**Results:**

A total of 229 mother-child pairs (mean [SD] maternal age, 29.45 [5.67] years; 116 female children [50.7%] and 113 male children [49.3%]) who had MUF_SG_ measured were included in the study. Median (IQR) MUF_SG_ was 0.76 (0.51-1.19) mg/L, and 32 participants (14.0%) had a Total Problems T score in the borderline clinical or clinical range. A 1-IQR (0.68 mg/L) increase in MUF_SG_ was associated with nearly double the odds of the Total Problems T score being in the borderline clinical or clinical range (odds ratio, 1.83; 95% CI, 1.17-2.86; *P* = .008), as well as with a 2.29-point increase in T score for the Internalizing Problems composite (B = 2.29; 95% CI, 0.47-4.11; *P* = .01) and a 2.14-point increase in T score for the Total Problems composite (B = 2.14; 95% CI, 0.29-3.98; *P* = .02).

**Conclusions and Relevance:**

In this prospective cohort study of mother-child pairs in Los Angeles, California, prenatal fluoride exposure was associated with increased neurobehavioral problems. These findings suggest that there may be a need to establish recommendations for limiting fluoride exposure during the prenatal period.

## Key Points

**Question:**

Is prenatal fluoride exposure associated with child neurobehavior in a US-based sample?

**Findings:**

In this cohort study of 229 pregnant women and their children, a 0.68 mg/L (ie, 1 IQR) increase in specific gravity–adjusted maternal urinary fluoride during pregnancy was associated with nearly double the odds of T scores for total child neurobehavioral problems being in the borderline clinical or clinical range.

**Meaning:**

These findings suggest that prenatal fluoride exposure may increase risk of neurobehavioral problems among children living in an optimally fluoridated area in the US.

## Teaser

This cohort study analyzes associations of specific gravity–adjusted maternal urinary fluoride with child neurobehavioral development at 36 months among a cohort of individuals in Los Angeles, California.

## Introduction

Fluoride levels in community drinking water systems in the US have been adjusted to prevent dental caries since 1945.^1^ Currently, 73% of the US receives fluoridated water at a targeted concentration of 0.7 mg/L (to convert to millimoles per liter, multiply by 0.05263). This has been considered optimal for preventing dental caries while minimizing risk of adverse systemic health effects.^2^ Most of Los Angeles County, California is at least partially fluoridated.^3,4^ Fluoride can also naturally occur in soil and rock or be released into the environment via industrial processes.^5,6^

It is widely established that exposure to high fluoride levels can adversely affect neurodevelopment^7^; however, findings from recent studies conducted in Mexico and Canada^8,9,10,11^ suggest that fluoride exposure at lower US-relevant levels may also be associated with poorer neurodevelopment. Specifically, higher prenatal fluoride exposure in Canada and/or Mexico has been associated with lower IQ among children aged 3 to 4 years in Canada^10^ and children aged 6 to 12 years in Mexico,^9^ increased symptoms of attention-deficit/hyperactivity disorder (ADHD) among children aged 6 to 12 years,^12^ poorer executive function among children aged 3 to 5 years,^13^ and poorer performance on measures of global cognition among 12- and 24-month-old boys.^14^ A recent systematic review conducted by the National Toxicology Program reported “with moderate confidence that higher fluoride exposure…is consistently associated with lower IQ in children.”^15^ The report^15^ also highlighted the lack of US studies investigating associations of fluoride exposure with neurodevelopment or cognition and stated that US studies would be valuable. To our knowledge, we conducted the first, US-based study to examine associations of prenatal fluoride exposure with child neurobehavioral outcomes.

## Methods

### Study Design and Participants

This cohort study was approved by the institutional review boards at The University of Southern California and The University of Florida and followed the Strengthening the Reporting of Observational Studies in Epidemiology (STROBE) reporting guideline. This study included mother-child pairs from the Maternal and Developmental Risks from Environmental and Social Stressors (MADRES) cohort.^16^ MADRES is a prospective pregnancy cohort consisting of 1065 predominately Hispanic women of low socioeconomic status residing in urban Los Angeles, California.^16^ Briefly, in 2015, pregnant women were recruited from prenatal care clinicians in Los Angeles serving predominantly medically underserved communities and provided written informed consent. Eligibility criteria include being 18 years of age or older, less than 30 weeks’ gestation at the time of recruitment, and being able to speak English or Spanish fluently. Exclusion criteria included having a multiple gestation pregnancy; being HIV positive; having a physical, mental, or cognitive disability that would prevent participation or provision of informed consent; and current incarceration.^16^ The current study included mother-child pairs from the MADRES prospective cohort who had maternal urinary fluoride (MUF) measured during the third trimester of pregnancy and child scores on the Preschool Child Behavior Checklist (CBCL) for ages 1.5 to 5 years at age 36 months (eFigure 1 in Supplement 1).

### Exposure

#### Urinary Fluoride

Single spot urine samples were collected from MADRES participants during the third trimester of pregnancy (from 2017-2020). The mean (range) gestational age at third trimester urine collection was 31.6 (26.9-36.0) weeks. MUF was measured at the Oral Health Research Institute at the Indiana University School of Dentistry using the Martinez Mier et al modification^17,18^ of the hexamethyldisiloxane microdiffusion method of Taves et al^19^ (see the eMethods in Supplement 1 for additional details). MUF measurements were adjusted for specific gravity (MUF_SG_). Urinary fluoride was utilized because it provides a reliable measure of total fluoride intake. It is also the most widely employed measure of individual fluoride exposure in epidemiological studies, including those assessing neurodevelopment.^10,11,12,20,21^

### Outcomes

We examined child neurobehavioral problems. These included internalizing and externalizing symptoms and symptoms consistent with *Diagnostic and Statistical Manual of Mental Disorders* (Fifth Edition [*DSM-5*]) diagnostic categories.

### Preschool CBCL for Ages 1.5 to 5 Years

Child neurobehavioral outcomes were assessed from 2020 to 2023 via the Preschool CBCL, a valid measure of neurobehavior.^22,23,24^ The Preschool CBCL is a parent-reported measure of 99 items that was administered in MADRES when the child was approximately 36 months old. Children were rated on the CBCL by their mothers. The CBCL is available in English and Spanish. CBCL scores comprise 7 syndrome scales (Emotionally Reactive, Anxious-Depressed, Somatic Complaints, Withdrawn, Sleep Problems, Attention Problems, and Aggressive Behavior) characterizing problems that tend to co-occur together. The CBCL also includes 5 *DSM-5*–oriented scales that are comprised of items determined to be consistent with *DSM-5* diagnostic categories (Depressive Problems, Anxiety Problems, Oppositional Defiant Problems, Autism Spectrum Problems, and ADHD Problems). Scores on CBCL syndrome scales are grouped to produce an Internalizing Problems composite score and Externalizing Problems composite score. Scales that focus primarily on issues within the self comprise the Internalizing Problems composite. Conversely, scales that focus on other-directed problems and expectations for the child comprise the Externalizing Problems composite. Lastly, a Total Problems composite score is calculated by summing scores on all 99 items.^24^ Internalizing Problems, Externalizing Problems, and Total Problems composite T scores range from 28 to 100. T scores ranging from 60 to 63 are in the borderline clinical range, whereas those above 63 are in the clinical range.^24^ We calculated 2-category clinical index variables of normal vs borderline clinical or clinical for statistical analyses for each composite variable (see the eMethods in Supplement 1 for additional details about the CBCL scales).

### Covariates

Covariates were selected using a directed acyclic graph (eFigure 2 in Supplement 1), and included maternal age (continuous), education (less than 12th grade, completed 12th grade, some college or technical school, completed college, and some graduate training), ethnicity by nativity (non-Hispanic, US-born Hispanic, or non–US-born Hispanic), marital status (decline to answer, married, living together, never married and single, divorced or separated, or widowed), prepregnancy body mass index (continuous; calculated as weight in kilograms divided by height in meters squared) and prenatal household income (unknown,<$15 000, $15 000-$29 999, $30 000-$49 999, $50 000-$99 999, and ≥$100 000), as well as child sex. Categories for ethnicity by nativity were defined by study principal investigators, and ethnicity was included because it has been shown to be associated with fluoride exposure and neurodevelopment. We adjusted for ethnicity as a proxy for structural racism rather than as a biological difference. We recoded marital status based on cohabitation status (eMethods in Supplement 1).

### Statistical Analysis

Descriptive statistics were calculated for MUF_SG_, sociodemographic variables, and scores on the CBCL. We conducted linear regression adjusted for covariates to examine associations of third trimester MUF_SG_ with CBCL composite T scores as well as raw scores on CBCL syndrome scales and *DSM-5*–oriented scales. Assumptions of linear regression were satisfied for models examining associations of MUF_SG_ with CBCL composite T scores; however, for several models examining associations of MUF_SG_ with CBCL syndrome scales and *DSM-5*–oriented scales, linear regression assumptions were not satisfied. Therefore, a natural logarithm transformation was applied and a constant of 1 was added (to account for scores of 0) to the raw scores for these scales to satisfy linear regression assumptions (see the eMethods in Supplement 1 for an expanded statistical analysis plan). We also tested whether child sex modified associations of MUF_SG_ with CBCL scores by including a MUF_SG_ × sex term in regression models to be retained if statistically significant. We conducted logistic regression examining associations of MUF_SG_ with binary clinical index variables. Additionally, in sensitivity analyses, we conducted Poisson regression with robust error variances to determine the relative risk of scoring in the normal compared with borderline clinical or clinical range for clinical index variables. We also conducted binary logistic regression that included 2-category clinical index dependent variables of nonclinical (ie, normal or borderline) vs clinical for each clinical index variable. We conducted several additional sensitivity analyses that are reported in the eMethods in Supplement 1. We excluded women who reported prenatal smoking (6 participants). Statistical analyses were performed using SPSS statistical software version 28 (IBM) and STATA/MP version 13.0 (Stata Corp). The criterion for statistical significance was an α < .05. Data analysis occurred from October 2022 to March 2024.

## Results

There were 229 mother-child pairs (mean [SD] maternal age, 29.45 [5.67] years; 116 female children [50.7%] and 113 male children [49.3%]) included in this study. See Table 1 for sociodemographic characteristics and exposure variables. For a comparison of sociodemographic characteristics between the current study sample and overall MADRES cohort with a live birth, see eTable 1 in Supplement 1. Most participants (192 participants) reported fasting in the third trimester for at least 8 hours. MUF_SG_ did not differ between women who reported fasting and those who did not. Median (IQR) MUF_SG_ was 0.76 (0.51-1.19) mg/L. Mean (SD) T scores were 47.69 (11.60) for the Total Problems composite, 47.13 (11.62) for the Internalizing Problems composite, and 46.48 (10.68) for the Externalizing Problems composite (Figure). Of all participants, 32 (14.0%) had a Total Problems T score in the borderline clinical or clinical range, 35 (15.3%) had an Internalizing Problems T score in the borderline clinical or clinical range, and 23 (10.0%) had an Externalizing Problems T score in the borderline clinical or clinical range. Descriptive statistics for CBCL syndrome and DSM-oriented scale raw scores are presented in eFigure 3, eFigure 4, and eTable2 in Supplement 1.

**Table 1.** Descriptive Statistics for Demographic and Exposure Variables

Participant characteristic	Participants, No. (%) (N = 229)	Third trimester MUF_SG_, median (IQR), mg/L
Maternal age at consent, mean (SD), y	29.45 (5.67)	NA
Child age at Preschool Child Behavior Checklist assessment, mean (range), mo	36.02 (35.12-37.62)	NA
Child sex		
Female	116 (50.7)	0.76 (0.51-1.22)
Male	113 (49.3)	0.76 (0.51-1.16)
Prepregnancy body mass index^a^		
Underweight (<18.5)	5 (2.2)	0.71 (0.45-1.83)
Normal (18.5-24.9)	60 (26.2)	0.78 (0.48-1.32)
Overweight (25.0-29.9)	80 (34.9)	0.78 (0.55-1.25)
Obese (≥30.0)	84 (36.7)	0.74 (0.50-1.08)
Household income, $		
Unknown	64 (27.9)	0.74 (0.43-1.17)
<15 000	41 (17.9)	0.78 (0.51-1.12)
15 000-29 000	55 (24.0)	0.68 (0.43-0.98)
30 000-49 999	35 (15.3)	0.81 (0.52-1.24)
50 000-99 999	15 (6.6)	0.72 (0.52-0.93)
≥100 000	19 (8.3)	1.46 (0.81-1.96)
Maternal education		
<High school	54 (23.6)	0.73 (0.49-1.20)
High school	65 (28.4)	0.76 (0.52-1.08)
Some college or technical school	61 (26.6)	0.73 (0.46-0.93)
4-y of College	31 (13.5)	0.86 (0.61-1.37)
Some graduate training	18 (7.9)	1.31 (0.68-1.88)
Maternal ethnicity by nativity^b^		
Non-Hispanic	44 (19.8)	1.04 (0.72-1.80)
Non-US-born Hispanic	96 (43.2)	0.76 (0.51-1.23)
US-born Hispanic	82 (36.9)	0.69 (0.43-0.99)
Habitation status		
Cohabitating	168 (83.4)	0.76 (0.51-1.25)
Not cohabitating	48 (21.0)	0.77 (0.47-1.09)
Missing or declined to answer	13 (5.7)	0.79 (0.52-1.08)
MUF_SG_, median (IQR), mg/dL		
First trimester^c^	0.64 (0.43-0.98)	NA
Third trimester	0.76 (0.51-1.19)	NA
Average across first and third trimester^d^	0.75 (0.52-1.11)	NA
First trimester blood lead level, median (IQR), μg/dL^e^	0.36 (0.26-0.60)	NA

Abbreviations: MUF_SG_, maternal urinary fluoride adjusted for specific gravity; NA, not applicable.

SI Conversions: To convert fluoride to millimoles per liter, multiply by 0.05263; lead to micromoles per liter, multiply by 0.0483.

^a^ Body mass index was calculated as weight in kilograms divided by height in meters squared.

^b^ Based on 222 participants due to missing data for this variable.

^c^ Based on 155 participants.

^d^ Based on 154 participants.

^e^ Based on 123 participants.

**Figure.**
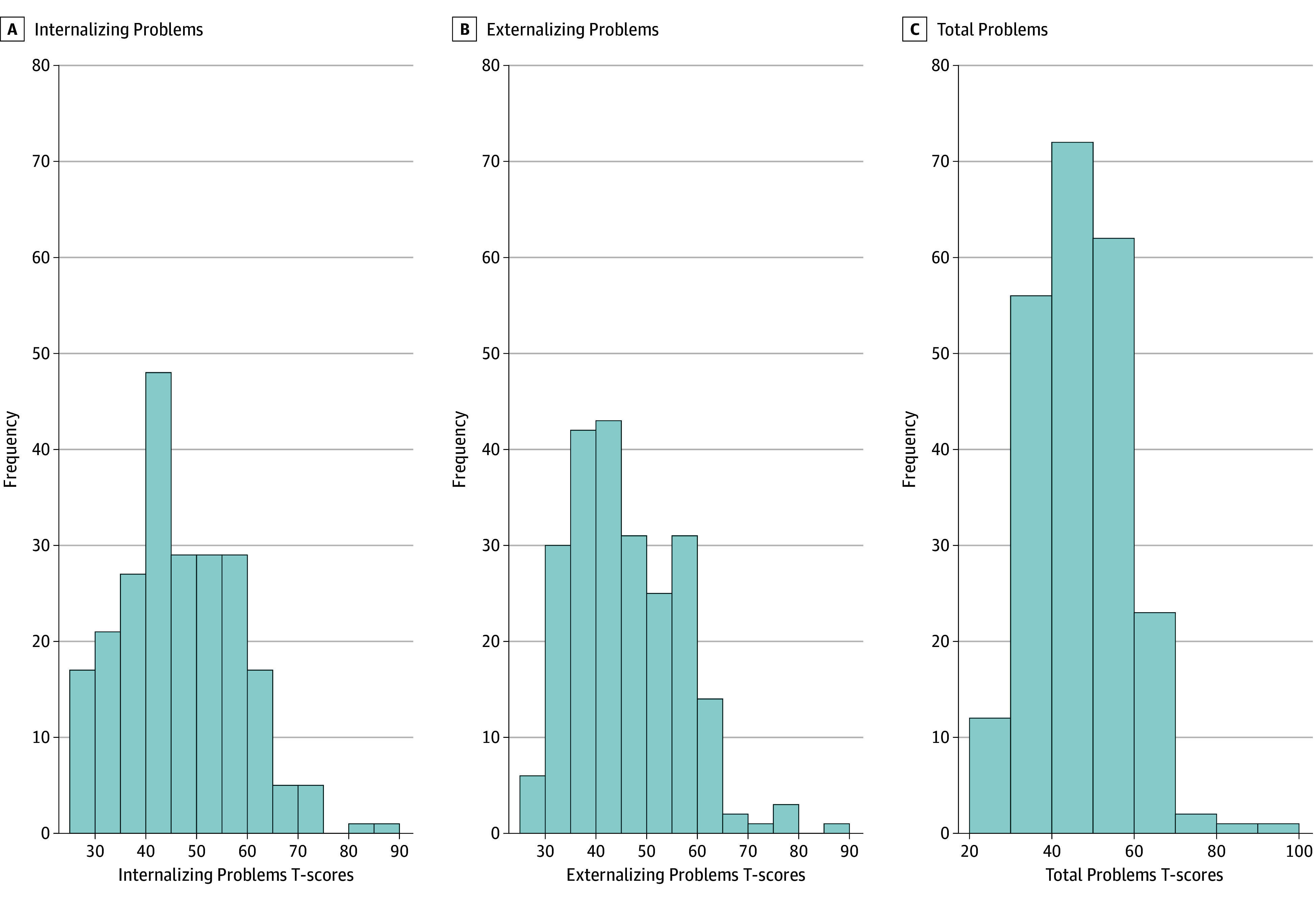
Distributions of T Scores for the Preschool Child Behavior Checklist Index Scales Among Children in the Maternal and Developmental Risks From Environmental and Social Stressors Cohort at Age 3 Years The blue histogram bars depict the frequency of T scores among participants per Child Behavior Checklist index scale including the internalizing problems scale (A), externalizing problems scale (B), and total problems scale (C). Frequencies and percentages are based on a total of 229 participants.

Associations of MUF_SG_ with CBCL composite T scores and binary clinical index variables are presented in Table 2. A 1-IQR (0.68 mg/L) increase in MUF_SG_ was associated with nearly double the odds of having a Total Problems T score in the borderline clinical or clinical range compared with the normal range (odds ratio [OR], 1.83; 95% CI, 1.17-2.86; *P* = .008). Additionally, a 1-IQR increase in MUF_SG_ was associated with a 2.29-point increase in Internalizing Problems T scores (B = 2.29; 95% CI, 0.47-4.11; *P* = .01) and 2.14-point increase in Total Problems T scores (B = 2.14; 95% CI, 0.29-3.98; *P* = .02). Associations of MUF_SG_ with Externalizing Problems T scores or odds of having an Internalizing Problems T score in the borderline clinical or clinical range compared with the normal range were also positive but not statistically significant (Table 2). Risk ratios were generally consistent with these ORs; however, magnitudes were smaller, and the *P* value for the risk ratio for the Internalizing Problems binary clinical index variable was statistically significant (eTable 3 in Supplement 1). Sensitivity analyses that included nonclinical vs clinical index dependent variables were also consistent (eTable 4 in Supplement 1).

**Table 2.** Associations of MUF_SG_ in the Third Trimester With CBCL Composite T Scores and Clinical Composite Index Variable Category^a^

CBCL composite scale^b^	Composite T scores		Clinical index variable scores	
	B (95% CI)	*P* value	OR (95% CI)^c^	*P* value
Total Problems	2.14 (0.29 to 3.98)	.02^d^	1.83 (1.17 to 2.86)	.008^d^
Internalizing Problems	2.29 (0.47 to 4.11)	.01^d^	1.48 (0.96 to 2.30)	.08
Externalizing Problems	1.45 (−0.27 to 3.17)	.10	1.38 (0.83 to 2.28)	.21

Abbreviations: CBCL, Preschool Child Behavior Checklist; MUF_SG_, maternal urinary fluoride adjusted for specific gravity; OR, odds ratio.

^a^ Calculations are based on a total of 229 participants. B values and ORs are presented according to an IQR (ie, 0.68 mg/L [to convert to millimoles per liter, multiply by 0.05263]) increase in MUF_SG_. Analyses were adjusted for maternal age, prepregnancy body mass index, ethnicity by nativity, maternal education, household income, maternal cohabitation, and child sex.

^b^ For clinical composite index scores, normal = 0 and borderline clinical or clinical = 1. Higher CBCL scores indicate more symptoms of neurobehavioral problems.

^c^ ORs reflect the odds of having a score in the borderline clinical or clinical range compared with the normal range.

^d^ Denotes statistical significance.

Associations of MUF_SG_ with raw scores for CBCL syndrome scales and *DSM-5*–oriented scales are presented in Table 3. A 1-IQR increase in MUF_SG_ was associated with a 13.54% increase in raw scores for the Emotionally Reactive CBCL syndrome scale (B = 0.13; 95% CI, 0.02-0.24; *P* = .02), and a 19.60% increase in raw scores for the Somatic Complaints CBCL syndrome scale (B = 0.18; 95% CI, 0.07-0.28; *P* = .001). Additionally, a 1-IQR increase in MUF_SG_ was associated with an 11.29% increase in scores on the *DSM-5*–oriented Anxiety Problems scale of the CBCL (B = 0.11; 95% CI, 0.003-0.21; *P* = .045) and an 18.53% increase in scores on the *DSM-5*–oriented Autism Spectrum Problems scale of the CBCL (B = 0.17; 95% CI, 0.04-0.30; *P* = .009). There were no other associations of MUF_SG_ with other syndrome scales or *DSM-5*–oriented scales. There was no interaction between fluoride and sex.

**Table 3.** Associations of MUF_SG_ in the Third Trimester With Raw Scale Scores^a^

Scale	B (95% CI)	*P* value	Increase in CBCL score per IQR increase in MUF_SG_, %
Natural log transformed			
CBCL syndrome scale			
Emotionally Reactive	0.13 (0.02 to 0.24)	.02^b^	13.54
Anxious-Depressed	0.08 (−0.03 to 0.19)	.15	8.22
Somatic Complaints	0.18 (0.07 to 0.28)	.001^b^	19.60
Withdrawn	0.09 (−0.03 to 0.21)	.14	9.20
Sleep Problems	0.07 (−0.05 to 0.18)	.25	6.82
Attention Problems	0.06 (−0.05 to 0.16)	.28	5.76
Aggressive Behavior	0.10 (−0.03 to 0.24)	.14	10.96
CBCL *DSM-5*–oriented scale			
Depressive Problems	0.09 (−0.02 to 0.20)	.10	9.53
Anxiety Problems	0.11 (0.003 to 0.21)	.045^b^	11.29
Oppositional Defiant Problems	0.08 (−0.04 to 0.19)	.20	8.00
Autism Spectrum Problems	0.17 (0.04 to 0.30)	.009^b^	18.53
Not natural log tranformed, *DSM-5*–oriented scale: ADHD Problems	0.41 (−0.07 to 0.88)	.10	Point increase, 0.41

Abbreviations: ADHD, attention-deficit/hyperactivity disorder; CBCL, Preschool Child Behavior Checklist; *DSM-5*, *Diagnostic and Statistical Manual of Mental Disorders* (Fifth Edition); MUF_SG_, maternal urinary fluoride adjusted for specific gravity.

^a^ Calculations are based on a total of 229 participants. B coefficients are presented according to an IQR (ie, 0.68 mg/L [to convert to millimoles per liter, multiply by 0.05263]) increase in MUF_SG_. CBCL scales were natural log-transformed plus a constant of 1 to satisfy assumptions of linear regression, except for the *DSM-5* ADHD problems scale because linear regression assumptions were satisfied for that model. Analyses were adjusted for maternal age, prepregnancy body mass index, ethnicity by nativity, maternal education, household income, maternal cohabitation, and child sex. Higher CBCL scores indicate more symptoms of neurobehavioral problems.

^b^ Denotes statistical significance.

MUF_SG_ during the first trimester was also positively associated with CBCL scores (eTable 5 and eTable 6 in Supplement 1) and when first trimester blood lead level was included as a covariate in sensitivity analyses, the magnitudes of associations became larger and previously nonsignificant findings became significant associations in models for both the first and third trimester (eTables 7-9 in Supplement 1). Associations of MUF_SG_ with CBCL scores in the third trimester remained generally the same when examined among only the sample of women who fasted for at least 8 hours (192 participants) and when adjusting for maternal smoking during pregnancy (eTables 10-13 in Supplement 1). Lastly, magnitudes of associations of mean MUF_SG_ across the first and third trimesters with CBCL scores were larger than associations of MUF_SG_ in only the third trimester with CBCL scores (eTable 14 and eTable 15 in Supplement 1).

## Discussion

To our knowledge, this is the first US-based cohort study to examine associations of prenatal fluoride exposure with child neurobehavior. The study sample resided in a predominately fluoridated region and had fluoride exposures that are typical of those living in fluoridated communities in North America.^17,25,26^ For example, Till et al^25^ reported a median MUF_SG_ of 0.77 mg/L among women living in fluoridated communities in Canada. We found that women with higher fluoride exposure during pregnancy tended to rate their children higher on overall neurobehavioral problems and internalizing symptoms, including emotional reactivity, anxiety, and somatic complaints by age 3 years. Furthermore, each 0.68 mg/L increase in MUF_SG_ was associated with nearly double the odds of total neurobehavioral problems being in the borderline clinical or clinical range. Women with higher MUF_SG_ during pregnancy also tended to rate their children higher on Autism Spectrum Disorder symptoms. The effect sizes observed in this study are sizable considering the relatively low urinary fluoride levels of participants.

Findings from this study are consistent with a recent Canadian study^13^ of over 600 maternal-child pairs in the Calgary cohort of the Alberta Pregnancy Outcomes and Nutrition study. The study found that exposure to drinking water fluoridated at 0.7 mg/L throughout pregnancy was associated with symptoms of executive dysfunction, including poorer inhibitory control, and decreased cognitive flexibility among children aged 3 to 5 years. However, associations were most pronounced among girls.^13^ Although we did not observe sex-specific associations in the current study, higher MUF_SG_ was associated with higher symptoms of Autism Spectrum Disorder and anxiety, which are also associated with poorer cognitive flexibility.^27,28,29^ Another recent study^12^ conducted in the Early Life Exposures in Mexico to Environmental Toxicants (ELEMENT) cohort found that higher creatinine-adjusted MUF was associated with higher scores on measures of inattention and overall ADHD symptoms from ages 6 to 12 years. While we did not find associations of MUF_SG_ with symptoms of inattention or ADHD, this may reflect the timing of neurobehavioral assessment because symptoms of inattention are more difficult to assess (and ADHD is more difficult to diagnose) in children younger than 4 years. Although no other prospective studies, to our knowledge, have examined associations of prenatal fluoride exposure with CBCL scores, a recent cross-sectional study^30^ of 12-year-old children in the Cincinnati Childhood Allergy and Air Pollution Study found that higher specific gravity-adjusted urinary fluoride levels were associated with higher somatic symptoms scores and odds of internalizing T scores being in a clinically at-risk range (defined as a T score ≥60) on the Behavior Assessment System for Children (Second Edition), particularly among boys. Still, an earlier study of 7- to 11-year-old children residing in Boston^31^ found no association of dental fluorosis or environmental fluoride exposure (assessed via questionnaire) with parent-reported neurobehavioral problems on the CBCL.

Other studies conducted in Canada and Mexico have found associations of higher prenatal fluoride exposure at US-population–relevant levels with poorer neurocognitive outcomes, including lower IQ.^8,9,10,12,14,21^ For example, a study conducted in the ELEMENT cohort found that each 0.5 mg/L increase in creatine-adjusted MUF was associated with a more than 2-point reduction in global cognitive functioning or IQ across 3 time points during middle to late childhood.^21^ Similarly, research conducted in the Canadian Maternal-Infant Research on Environmental Chemicals cohort found that each 1 mg/L increase in MUF_SG_ was associated with a 4.49-point lower IQ score in boys.^8,10^ Taken together, the weight of the scientific literature supports an association of prenatal fluoride exposure with adverse child cognitive and neurobehavioral development in North America. Still, when considering the global body of scientific literature, there are some inconsistencies.^32,33,34^

It is well-established that the prenatal and early postnatal periods are windows of susceptibility for neurodevelopmental impacts of environmental toxicant exposures.^35,36^ Animal studies have delineated potential mechanisms underlying the association of prenatal fluoride exposure with neurobehavioral development. A 2022 study^37^ found that at 90 days of age, male rats who were prenatally and perinatally exposed to relatively low fluoride levels exhibited altered neurobiochemical markers of oxidative damage, glutamate metabolism, and acetylcholinesterase activity. Another recent study^38^ found that at 90 days of age, female rats exposed to low fluoride levels during gestation and lactation exhibited decreased messenger RNA expression of the α7 nicotinic acetylcholine receptor (α7nAChR) and reduced hippocampal catalase activity (an indicator of oxidative stress). Neurochemical changes observed in both studies^37,38^ have been replicated in other animal as well as in vitro studies that included high fluoride exposures.^39,40,41^ Interestingly, both oxidative stress and alterations of the α7nAChR in particular have been implicated in the pathophysiology of neurodevelopmental disorders, including Autism Spectrum Disorder.^42,43^ Furthermore, alterations in glutamate pathways have been implicated in the cause and treatment of anxiety disorders.^44^ Prenatal fluoride exposure may also adversely affect neurodevelopment and cognition by causing mitochondrial dysfunction which can increase oxidative stress, blocking autophagosome-lysosome fusion which can contribute to cellular damage, and by causing synaptic dysfunction.^45,46,47^ Additionally, prenatal fluoride exposure, even at low levels, can suppress maternal thyroid gland activity which can contribute to cognitive and neurobehavioral problems in offspring.^48,49^

### Strengths and Limitations

There are notable strengths of the current study, including the use of individual biomarker measures of exposure assessment that provide an estimate of fluoride intake from all sources, and the adjustment for a breadth of covariates associated with fluoride exposure, metabolism, and neurodevelopment. Additionally, our study addressed a limitation of prior studies on fluoride exposure and neurodevelopment by including a sample of predominately fasting pregnant women, which can be difficult to achieve. However, there are also limitations. First, we measured fluoride in spot samples rather than 24-hour urine samples, which can be influenced by daily behaviors (eg, food and beverage consumption or use of fluoridated dental products), and therefore increase random error. Still, the inclusion of mostly fasting urine samples reduces the potential impact of food and beverage consumption on urinary fluoride concentrations. Second, we were limited in our ability to examine patterns of associations of fluoride exposure with neurobehavior according to trimester because only a subsample of participants had urine available for fluoride analyses in the first trimester and most participants did not fast prior to urine collection. Nevertheless, associations of first trimester MUF_SG_ with CBCL scores after adjusting for blood lead were in the same direction as for the third trimester. Third, we did not have data on tap water consumption habits for the study sample; however, home cooking rates were high, and rice tended to be a dietary staple among MADRES participants, which can be a source of tap water fluoride exposure. Fourth, given that the study sample resided in Los Angeles, California, and was predominately Hispanic, we do not know whether findings observed in this study are generalizable to other US populations or are nationally representative. Fifth, this study excluded participants who delivered their babies prior to 30 weeks’ gestation which precluded examination of associations of MUF_SG_ with neurobehavior among children who were born very preterm. Sixth, lead concentrations in whole blood were only measured for most of the study sample during the first trimester, and therefore we were only able to adjust for first trimester blood lead in our third trimester analyses. Still, we do not anticipate confounding of associations of MUF_SG_ with CBCL scores by blood lead given that the inclusion of first trimester blood lead in first and third trimester models increased the magnitude of the associations. Furthermore, blood lead has been shown to be stable between the first and third trimesters of pregnancy,^50^ which supports the use of first trimester blood lead as a proxy for third trimester blood lead.

## Conclusions

This cohort study found that prenatal fluoride exposure was associated with increased risk for neurobehavioral problems among children residing in the US. These findings suggest that there may be a need to establish recommendations for limiting exposure to fluoride from all sources during the prenatal period, a time when the developing brain is known to be especially vulnerable to injury from environmental insults.

## Notes

Supplement 1. **eFigure 1.** Participant Selection Flowchart for the Current Study Sample
eMethods.
**eFigure 2.** Directed Acyclic Graph (DAG) for Covariate Selection **eTable 1.** Maternal Demographics According to Fluoride Sample **eFigure 3.** Distributions of Raw Scores for CBCL Syndrome Scales Among Children in the MADRES Study at Age 3; n = 229 **eFigure 4.** Distributions of Raw Scores for CBCL DSM-Oriented Scales Among Children in the MADRES Study at Age 3; n = 229 **eTable 2.** CBCL Scores at Age 36-Months in the MADRES Cohort **eTable 3.** Poisson Regression Estimating the Risk Ratio for Third Trimester MUFsg in Relation to CBCL Clinical Index Scores **eTable 4.** Sensitivity Analysis Including “Borderline Clinical” with “Non-Clinical” Group as the Reference in Logistic Regression of Trimester 3 MUFsg With CBCL Clinical Index Scores **eTable 5.** Sensitivity Analysis of Associations of MUFsg in Trimester 1 With CBCL Clinical Index Scores **eTable 6.** Sensitivity Analysis of Associations of Trimester 1 MUFsg With Composite T-Scores, and Syndrome or DSM Scale Raw CBCL Scores at Age 3 **eTable 7.** Associations of MUFsg in Trimester 1 with CBCL Scores Adjusting for Blood Lead **eTable 8.** Associations of MUFsg in Trimester 3 With CBCL Scores Adjusting for Trimester 1 Blood Lead **eTable 9.** Associations of MUFsg in Trimester 3 with CBCL Clinical Index Scores Adjusting for Trimester 1 Blood Lead **eTable 10.** Associations of MUFsg in Trimester 3 with CBCL Scores Among Women Who Fasted for ≥ 8 Hours **eTable 11.** Trimester 3 MUFsg in Relation to CBCL Clinical Index Scores Among Women who Fasted for ≥ 8 Hours **eTable 12.** Associations of MUFsg in Trimester 3 with CBCL Scores Including Women Who Smoked During Pregnancy **eTable 13.** Trimester 3 MUFsg in Relation to CBCL Clinical Index Scores Including Women Who Smoked During Pregnancy **eTable 14.** Associations of Average MUFsg Across Trimesters 1 and 3 With CBCL Scores **eTable 15.** Average MUFsg Across Trimesters 1 and 3 in Relation to CBCL Clinical Index Scores

Supplement 2.
Data Sharing Statement
