# Error in Figure

**DOI:** 10.1001/jamanetworkopen.2024.24057

**Published:** 2024-06-28

In the Original Investigation titled “Maternal Urinary Fluoride and Child Neurobehavior at Age 36 Months,”^1^ published May 20, 2024, there were errors in the Figure, including mislabeled y-axes (which should have been “Frequency” rather than “Frequency, %”), a mislabeled x-axis in panel B (which should have been “Externalizing Problems T-scores” rather than “Internalizing Problems T-Scores”), and a shifted y-axis scale that made the frequencies in the histogram appear incorrect. This article has been corrected.^1^
