# Supplementary material for: Maternal Urinary Fluoride and Child Neurobehavior at Age 36 Months
Source: JAMA Netw Open. 2024 May 20;7(5):e2411987. doi: 10.1001/jamanetworkopen.2024.11987 (PMC11107298; doi:10.1001/jamanetworkopen.2024.11987)
Supplement: Supplement 1. — eFigure 1. Participant Selection Flowchart for the Current Study Sample eMethods. eFigure 2. Directed Acyclic Graph (DAG) for Covariate Selection eTable 1. Maternal Demographics According to Fluoride Sample eFigure 3. Distributions of Raw Scores for CBCL Syndrome Scales Among Children in the MADRES Study at Age 3; n = 229 eFigure 4. Distributions of Raw Scores for CBCL DSM-Oriented Scales Among Children in the MADRES Study at Age 3; n = 229 eTable 2. CBCL Scores at Age 36-Months in the MADRES Cohort eTable 3. Poisson Regression Estimating the Risk Ratio for Third Trimester MUFsg in Relation to CBCL Clinical Index Scores eTable 4. Sensitivity Analysis Including “Borderline Clinical” with “Non-Clinical” Group as the Reference in Logistic Regression of Trimester 3 MUFsg With CBCL Clinical Index Scores eTable 5. Sensitivity Analysis of Associations of MUFsg in Trimester 1 With CBCL Clinical Index Scores eTable 6. Sensitivity Analysis of Associations of Trimester 1 MUFsg With Composite T-Scores, and Syndrome or DSM Scale Raw CBCL Scores at Age 3 eTable 7. Associations of MUFsg in Trimester 1 with CBCL Scores Adjusting for Blood Lead eTable 8. Associations of MUFsg in Trimester 3 With CBCL Scores Adjusting for Trimester 1 Blood Lead eTable 9. Associations of MUFsg in Trimester 3 with CBCL Clinical Index Scores Adjusting for Trimester 1 Blood Lead eTable 10. Associations of MUFsg in Trimester 3 with CBCL Scores Among Women Who Fasted for ≥ 8 Hours eTable 11. Trimester 3 MUFsg in Relation to CBCL Clinical Index Scores Among Women who Fasted for ≥ 8 Hours eTable 12. Associations of MUFsg in Trimester 3 with CBCL Scores Including Women Who Smoked During Pregnancy eTable 13. Trimester 3 MUFsg in Relation to CBCL Clinical Index Scores Including Women Who Smoked During Pregnancy eTable 14. Associations of Average MUFsg Across Trimesters 1 and 3 With CBCL Scores eTable 15. Average MUFsg Across Trimesters 1 and 3 in Relation to CBCL Clinical Index Scores [file jamanetwopen-e2411987-s001.pdf]

Supplemental Online Content

Malin AJ, Eckel SP, Hu H, et al. Maternal urinary fluoride and child neurobehavior at age 36-months. *JAMA Netw Open*. 2024;7(5):e2411987. doi:10.1001/jamanetworkopen.2024.11987

eFigure 1. Participant Selection Flow Chart for the Current Study Sample
eMethods
eFigure 2. Directed Acyclic Graph (DAG) for Covariate Selection
eTable 1. Maternal Demographics According to Fluoride Sample
eFigure 3. Distributions of Raw Scores for CBCL Syndrome Scales Among Children in the MADRES Study at Age 3; n=229
eFigure 4. Distributions of Raw Scores for CBCL DSM-Oriented Scales Among Children in the MADRES Study at Age 3; n=229
eTable 2. CBCL Scores at Age 36-Months in the MADRES Cohort
eTable 3. Poisson Regression Estimating the Risk Ratio for Third Trimester MUFsg in Relation to CBCL Clinical Index Scores
eTable 4. Sensitivity Analysis Including "Borderline Clinical" with "Non-Clinical" Group as the Reference in Logistic Regression of Trimester 3 MUFsg With CBCL Clinical Index Scores
eTable 5. Sensitivity Analysis of Associations of MUFsg in Trimester 1 With CBCL Clinical Index Scores
eTable 6. Sensitivity Analysis of Associations of Trimester 1 MUFsg With Composite *T*-Scores, and Syndrome or DSM Scale Raw CBCL Scores at Age 3
eTable 7. Associations of MUFsg in Trimester 1 with CBCL Scores Adjusting for Blood Lead
eTable 8. Associations of MUFsg in Trimester 3 With CBCL Scores Adjusting for Trimester 1 Blood Lead
eTable 9. Associations of MUFsg in Trimester 3 with CBCL Clinical Index Scores Adjusting for Trimester 1 Blood Lead
eTable 10. Associations of MUFsg in Trimester 3 with CBCL Scores Among Women Who Fasted for >= 8 Hours
eTable 11. Trimester 3 MUFsg in Relation to CBCL Clinical Index Scores Among Women who Fasted for >= 8 Hours
eTable 12. Associations of MUFsg in Trimester 3 with CBCL Scores Including Women Who Smoked During Pregnancy
eTable 13. Trimester 3 MUFsg in Relation to CBCL Clinical Index Scores Including Women Who Smoked During Pregnancy
eTable 14. Associations of Average MUFsg Across Trimesters 1 and 3 With CBCL Scores
eTable 15. Average MUFsg Across Trimesters 1 and 3 in Relation to CBCL Clinical Index Scores

This supplemental material has been provided by the authors to give readers additional information about their work.

© 2024 Malin AJ et al. *JAMA Network Open.*

eFigure 1. Participant Selection Flow Chart for the Current Study Sample

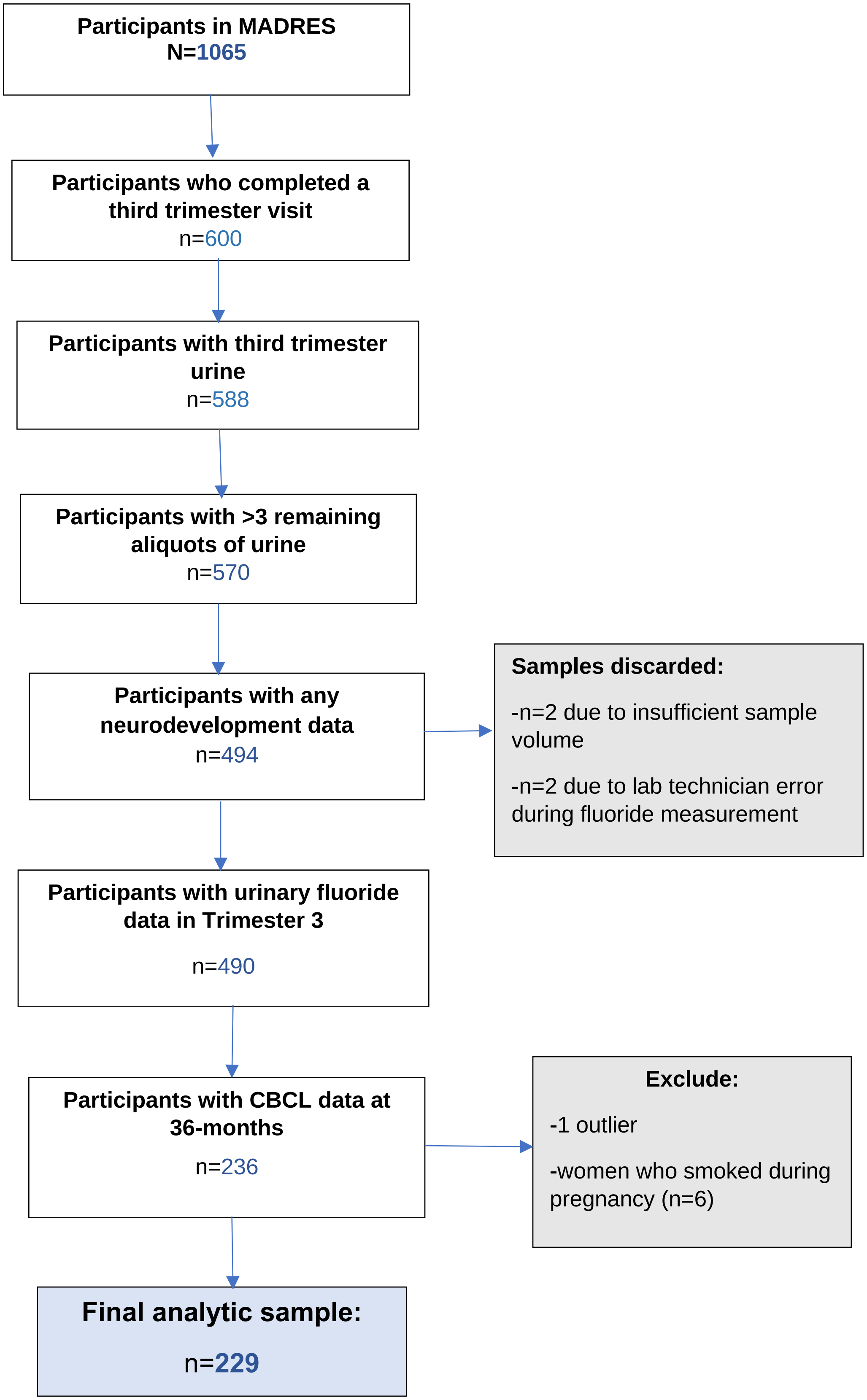

Legend. White boxes depict the flow of participant sample reduction from the overall MADRES cohort to the current study sample; grey boxes depict participants excluded from the current study sample; the blue box depicts the final analytic sample.

© 2024 Malin AJ et al. *JAMA Network Open.*

eMethods

Urine Collection and Fluoride Measurement

Urine samples were collected using 90-mL sterile specimen containers. They were transported on ice to the laboratory within 1 hour of collection. They were then aliquoted in 1.5 mL sterile cryovials (VWR) and stored at −80°C. Urinary fluoride was measured in 1mL of urine using a single 1.5 mL aliquot among participants who had > 3 aliquots of urine archived and any child neurodevelopment data. For urinary fluoride measurement, a standard fluoride curve was similarly prepared and used to determine the fluoride content of each sample. Analyses of all standards/samples were performed using a fluoride ion-specific electrode and a pH/lSE meter. Testing included a standard check, using a fluoride standard traceable to NIST, performed with daily sample analysis.

Specific Gravity Measurement

Urinary specific gravity was measured using a zero-setting calibrated ATAGO® Pen Refractometer under darkened conditions and was performed daily while setting up Petri dishes for fluoride analysis. Maternal urinary fluoride adjusted for specific gravity (MUFsg) was derived from the unadjusted fluoride value and specific gravity (SG) of each sample using the Levine Fahy equation: [$\text{Concentration}_{\text{SG normalized}} = \text{Concentration}_{\text{specimen}} (\text{SG}_{\text{reference}} - 1)/(\text{SG}_{\text{specimen}} - 1)$] where $\text{SG}_{\text{reference}}$ is the median SG for the cohort.[1] After fluoride analysis was complete, data were reviewed for quality assurance and approved.

Description of the Preschool Child Behavior Checklist (CBCL) 1.5-5

The CBCL consists of 99 items assessing child emotional and behavior problems that are rated from 0-2 based on the preceding 2 months.[2] The ratings for each item on the CBCL are defined as 0 = not true, 1 = somewhat or sometimes true, and 2 = very or often true. Several items also ask for descriptions of the problems.[2] CBCL scores comprise 7 syndrome scales and 5 DSM-oriented scales. The DSM scales have been shown to have clinical utility for differentiating neurodevelopmental disorders;[3] however, their unidimensionality is less clear; and thus findings pertaining to them should be interpreted more cautiously.[4] The CBCL syndrome scales include *Emotionally Reactive*, *Anxious-Depressed*, *Somatic Complaints*, *Withdrawn*, *Sleep Problems*, *Attention Problems*, and *Aggressive Behavior*. Emotionally Reactive, Anxious-Depressed, Somatic Complaints, and Withdrawn scales, comprise the Internalizing composite, while Attention Problems and Aggressive Behavior comprise the Externalizing composite. The DSM-Oriented scales included in the CBCL are *DSM Attention Deficit/Hyperactivity* (ADHD) *Problems*, *Depressive Problems*, *Anxiety Problems*, *Oppositional Defiant Problems, and Autism Spectrum Problems*.[2] For the current study, we used *T*-scores for composite scales and raw scores for syndrome and DSM-oriented scales in statistical analyses because *T*-scores for syndrome and DSM scales are truncated at 50, and therefore raw scores directly reflect differences in scores without influence by data transformations.[2]

The ranges for the CBCL composite *T*-scores are as follows: Internalizing, 29-100, Externalizing, 28-100, Total Problems, 28-100. The raw score ranges for the CBCL syndrome scales are as follows: Emotionally Reactive, 0-18; Anxious-Depressed, 0-16; Somatic Complaints, 0-22; Withdrawn, 0-16; Sleep Problems, 0-14; Attention Problems, 0-10; Aggressive Behavior, 0-38. The raw score ranges from the DSM-oriented scales are as follows: Depressive Problems, 0-20; Anxiety Problems, 0-20; Autism Spectrum Problems, 0-24; Attention Deficit/Hyperactivity Problems, 0-12; Oppositional Defiant Problems, 0-12.

Description of Covariates

Maternal Education. Participants were asked to report their last completed grade in school. Their responses were classified as less than 12th grade (i.e., did not finish high school), completed grade 12 (i.e., high school), some college or technical school, completed 4 years of college, and some graduate training after college.

Maternal Ethnicity by Nativity. Participants were classified as either non-Hispanic, US-Born Hispanic, or non-US-Born Hispanic based on their self-reported ethnicity and nativity.

Prenatal Household Income. Participants were asked during pregnancy, "in which of the following categories did your total household family income fall in the last year?" The categories included: don't know, less than $15,000, $15,000 to $29,999, $30,000 to $49,999, $50,000 to $99,999, $100,000 or more.

Marital Status. Participants were asked "What is your marital status?" The categories included: decline to answer, married, living together, never married and single, divorced or separated, or widowed.

© 2024 Malin AJ et al. *JAMA Network Open.*

Pre-pregnancy BMI. Pre-pregnancy weight was determined via self-report through interviewer-administered questionnaires during pregnancy. If missing, then the first weight of the index pregnancy (obtained from maternal electronic medical records) was used in lieu of self-reported pre-pregnancy weight. Height was measured during a prenatal visit at the 1st or 3rd trimester. Pre-pregnancy BMI was defined as [weight (kg) / height $(m)^2$]. Pre-pregnancy BMI was included as a continuous variable in statistical analyses.

Statistical Analysis

Recoding Categorical Covariates. We recoded covariates including maternal education, income, marital status, and ethnicity by nativity into zero-one indicator variables for regression analyses. For education, income, and ethnicity by nativity, we utilized existing categories; however, for marital status we collapsed categories according to cohabitation such that 1=living together or married, 2=never married, widowed, or divorced, and 3=decline to answer or missing. There were 10 participants with missing data for marital status and 3 participants who declined to answer the question. There were 7 participants with missing data for maternal ethnicity by nativity and they were designated to a "missing" category for regression analyses. There were no participants with missing data for household income, maternal education, or pre-pregnancy BMI.

Outliers. One deemed outlying datapoint with a third trimester MUFsg value of 7.99 mg/L was removed from statistical analyses. This value was more than 9 standard deviations from the mean. We deemed this datapoint to be an outlier warranting exclusion based on several factors. First, a MUFsg value of 7.99 mg/L in a North American population supplied with community drinking water is unlikely and indicative of atypical fluoride exposure prior to urine collection. Second, the first trimester MUFsg for this participant was 0.43 mg/L which is considerably lower and suggests that the third trimester MUFsg value of 7.99 mg/L is not representative of this participant's typical fluoride exposure. Third, at the outset of statistical analyses, we constructed generalized additive mixed (GAM) models to test for non-linear associations of MUFsg with CBCL composite variables (i.e., Total Problems, Internalizing, and Externalizing *T*-scores). We examined these models with and without the deemed outlying data point (i.e., third trimester MUFsg = 7.99 mg/L). Without the deemed outlying datapoint included the association of MUFsg with each of the CBCL composite variables was linear. However, the inclusion of the outlying datapoint skewed the entire model such that the associations became non-linear but only because of that one datapoint. Thus, inclusion of the outlying datapoint appeared to bias the shape of the association in the models in a manner that was not representative of the associations of MUFsg with CBCL composite *T*-scores for all other data points/participants.

Assumptions of Linear Regression. After excluding the deemed outlying datapoint, we conducted additional assessment of linearity in the models of MUFsg with CBCL scores. We examined scatterplots of third trimester MUFsg and CBCL scores fitted with loess curves which showed approximately linear associations. Furthermore, we tested cubic and quadratic terms in linear regression models. These were not statistically significant at $p < 0.05$ for most models, and therefore not retained. However, there were three scales for which slight quadratic trends were apparent and further explored. These scales were "Withdrawn", "Somatic Complaints" and "DSM-Anxiety Problems". However, when we compared model fit for quadratic versus linear models differences were minor (R square and standard error of estimate values were similar in magnitude). We decided to proceed with linear models as they appeared to provide a more holistic representation of the data.

We examined linear regression diagnostics to assess whether assumptions of linear regression were satisfied. In models testing associations of MUFsg with CBCL composite *T*-scores, assumptions were well satisfied. However, for models testing associations of MUFsg with CBCL syndrome and DSM-Oriented scales, the assumptions were not satisfied for most models, except for models examining associations of MUFsg with DSM-ADHD Problems (as well as other models in sensitivity analyses depending on the analysis). In the models for which the assumptions of linear regression were not satisfied, the residuals generated were not normally distributed, and variances appeared heterogenous. Thus, we applied a natural logarithm transformation and added a constant of 1 which helped to improve model fit and satisfy linear regression assumptions. Specifically, after this transformation was applied, residuals approximated a normal distribution and variances became more homogenous.

We examined variance inflation factors (VIF) and tolerance values to test for multicollinearity between variables and no issues were detected. Specifically, VIF values were all less than 5 and tolerance values all greater than 0.25. For logistic regression analyses of MUFsg with Internalizing, Externalizing, and Total problems binary clinical

index variables, we ran models with 100 iterations and results remained identical, which provides support for model stability.

Interpretations for measures of associations. For CBCL syndrome scales and all DSM-Oriented scales except for DSM-ADHD Problems, we applied a natural logarithm transformation and added a constant of 1. Additionally, results of linear regression for associations of MUFs with CBCL composite *T*-scores and syndrome and DSM-oriented scale raw scores were scaled to a given point change in CBCL score per IQR (i.e., 0.68 mg/L) increase in MUFsg. For logistic regression models, we calculated odds ratios that corresponded to a change in the odds of having a CBCL score in the borderline clinical or clinical range compared to the normal range according to an IQR (i.e., 0.68 mg/L) increase in MUFsg. We conducted additional calculations with the beta coefficients and confidence intervals (CIs) in linear and logistic regression models to scale these estimates according to an IQR (i.e., 0.68 mg/L) increase in MUFsg rather than a 1-point increase. For linear regression models, we multiplied the beta estimates and confidence intervals by 0.68. For logistic regression, we multiplied beta estimates by 0.68 and exponentiated them. To calculate confidence intervals according to an IQR increase in MUFsg, we took the natural logarithm of each the upper and lower bound of the 95% confidence interval for the odds ratio pertaining to a 1-point increase in MUFsg and multiplied each result by the IQR (i.e., 0.68). We then exponentiated each of these products:

$$\text{EXP}\ ((\text{LN}\ (95\%\text{CI Lower or Upper Bound})) * 0.68)$$

We interpret associations of MUFsg with CBCL scales for which we applied a natural logarithm transformation plus a constant of 1 as follows:

$$\log Y = b0 + b1*X + e$$
$$100*(1-e^{b1}) = \%\ \text{difference in Y per 1-unit change in X}$$

Additional Sensitivity Analyses. We examined associations of MUFsg during the first trimester with CBCL scores in a subsample of participants (n=155). These analyses are supplemental because most participants did not fast prior to urine collection during the first trimester, and the sample size was considerably smaller. We also adjusted for blood lead in trimesters one and three models given that exposure to fluoride and lead can co-occur. [5,6] We utilized first trimester blood lead since blood lead data were only available for most of our sample in trimester one. Nevertheless, correlations between blood lead concentrations during the first and third trimester were large (n=92, $\rho = 0.80$, $p < .001$). We conducted sensitivity analyses examining associations of MUFsg in Trimester 3 with CBCL scores among only women who fasted for at least 8-hours (n=192). We also conducted sensitivity analyses adjusting for maternal smoking during pregnancy rather than excluding women who reported smoking from the analysis. Lastly, we examined associations of average MUFsg across trimesters 1 and 3 with CBCL scores in a subsample with MUFsg at both timepoints (n=154). The correlation between trimesters 1 and 3 MUFsg was $\rho = .49$, $p < .001$.

References

1. Cone EJ, Caplan YH, Moser F, Robert T, Shelby MK, Black DL. Normalization of Urinary Drug Concentrations with Specific Gravity and Creatinine. *Journal of Analytical Toxicology*. 2009;33(1):1-7. doi:10.1093/jat/33.1.1
2. Achenbach TM, Rescorla, L.A. *Manual for the ASEBA Preschool Forms & Profiles*. 2000.
3. Cheng YL, Chu CL, Wu CC. How Do Children with Autism Spectrum Disorder and Children with Developmental Delays Differ on the Child Behavior Checklist 1.5-5 DSM-Oriented Scales? *Children (Basel)*. Jan 14 2022;9(1)doi:10.3390/children9010111
4. Neo WS, Suzuki T, Kelleher BL. Structural validity of the Child Behavior Checklist (CBCL) for preschoolers with neurogenetic syndromes. *Res Dev Disabil*. Feb 2021;109:103834. doi:10.1016/j.ridd.2020.103834
5. Mullenix PJ. A new perspective on metals and other contaminants in fluoridation chemicals. *Int J Occup Environ Health*. Apr-Jun 2014;20(2):157-66. doi:10.1179/2049396714y.0000000062
6. Maas RP, Patch SC, Christian AM, Coplan MJ. Effects of fluoridation and disinfection agent combinations on lead leaching from leaded-brass parts. *Neurotoxicology*. Sep 2007;28(5):1023-31. doi:10.1016/j.neuro.2007.06.006

© 2024 Malin AJ et al. *JAMA Network Open.*

eFigure 2. Directed Acyclic Graph (DAG) for Covariate Selection

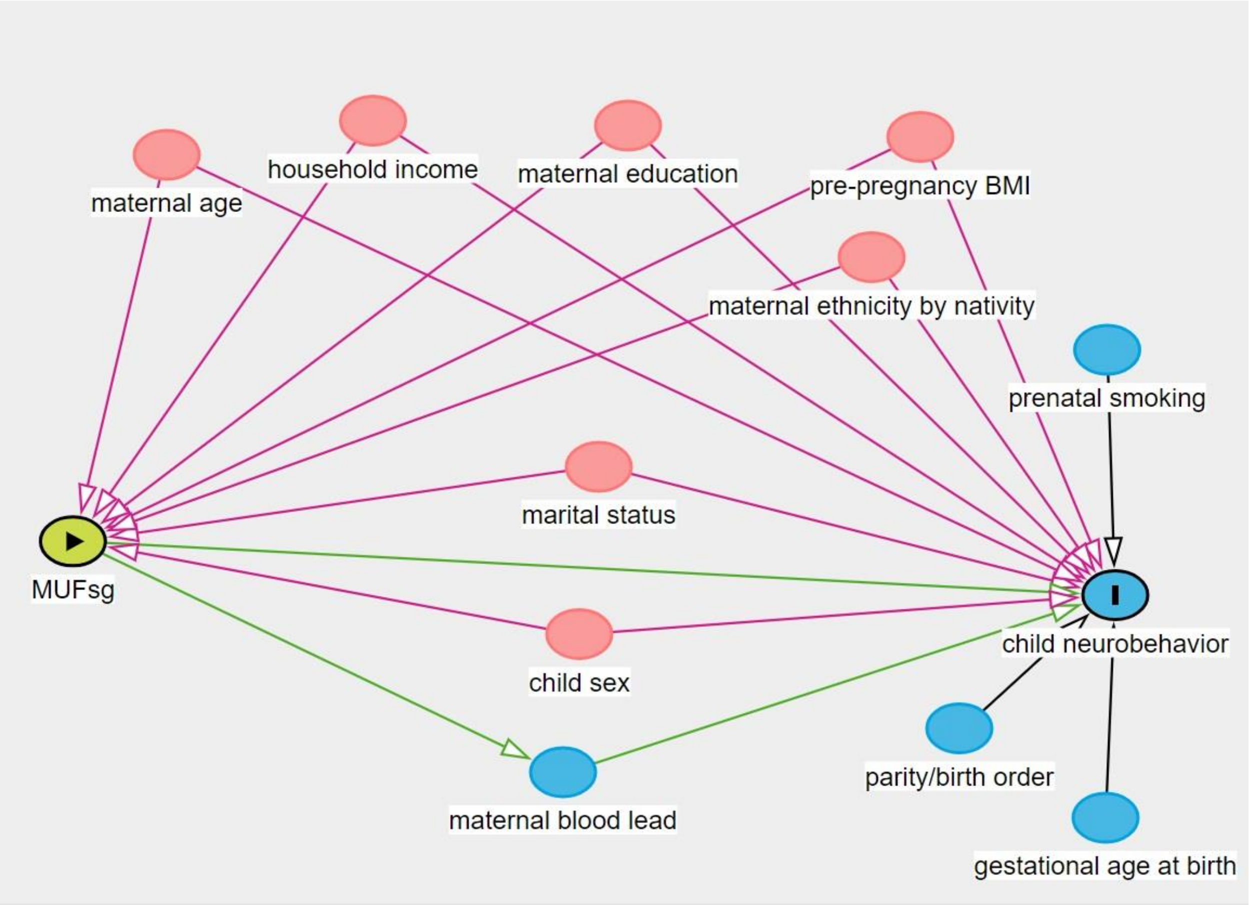

This figure was created using DAGitty. The exposure variable is depicted by a green circle with a rotated triangle; the outcome variable is depicted by a blue circle with a vertical line in the center; the pink circles connected by pink arrows depict covariates; the blue circle connected by green arrows depicts a potential mediating variable; blue circles connected to black arrows depict variables associated with the outcome.

© 2024 Malin AJ et al. *JAMA Network Open.*

eTable 1. **Maternal Demographics According to Fluoride Sample**

	Current Study Sample	Overall MADRES Cohort with a Live Birth
	N = 229	N = 883
**Maternal Age at Consent** (yrs.; Mean, SD)	29.45 (5.67)	28.33 (5.93)
**Child Sex** (freq., %) [a]		
Female	116 (50.7)	439 (50.3)
Male	113 (49.3)	434 (49.7)
**Pre-pregnancy BMI** (freq., %) [b]		
Underweight	5 (2.2)	22 (2.5)
Normal	60 (26.2)	262 (30.0)
Overweight	80 (34.9)	277 (31.8)
Obese	84 (36.7)	311 (35.6)
**Household Income** (freq., %) [c]		
Don't Know	64 (27.9)	267 (32.0)
< $15,000	41 (17.9)	193 (23.1)
$15,000 - $29,000	55 (24.0)	198 (23.7)
$30,000 to $49,999	35 (15.3)	95 (11.4)
$50,000 - $99,999	15 (6.6)	45 (5.4)
$100,000 or more	19 (8.3)	37 (4.4)
**Maternal Education** (freq., %) [c]		
< High School	54 (23.6)	209 (25.0)
High School	65 (28.4)	267 (32.0)
Some college/technical school	61 (26.6)	221 (26.5)
4-years of college	31 (13.5)	93 (11.1)
Some graduate training after college	18 (7.9)	45(5.4)
**Maternal Ethnicity by Nativity** [d] (freq., %)		
Non-Hispanic	44 (19.8)	165 (21.6)
Non-US-Born Hispanic	96 (43.2)	317 (41.5)
US-Born Hispanic	82 (36.9)	282 (36.9)
**Co-habitation Status**		
Cohabitating	168 (73.4)	511 (57.9)
Not Cohabitating	48 (21.0)	206 (23.3)
Missing or decline to answer	13 (5.7)	166 (18.8)

a. n=873, b. n=872, c. n=835, d. n=764 for overall sample

© 2024 Malin AJ et al. *JAMA Network Open.*

eFigure 3. Distributions of Raw Scores for CBCL Syndrome Scales Among Children in MADRES at Age 3

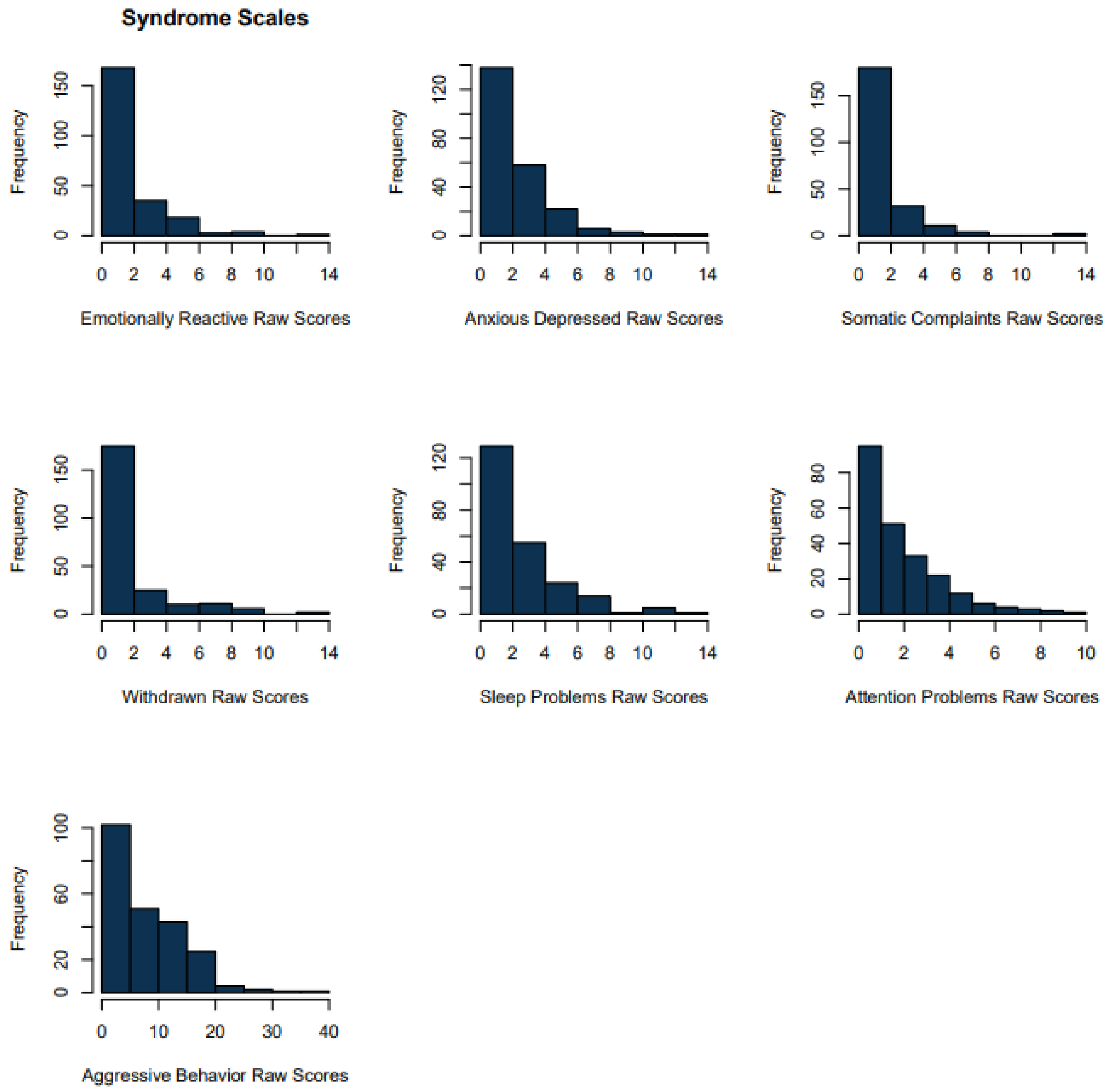

n=229; navy blue histogram bars depict the frequency of raw scores among participants per CBCL syndrome scale.

© 2024 Malin AJ et al. *JAMA Network Open.*

eFigure 4. Distributions of Raw Scores for CBCL DSM Scales Among Children in MADRES at Age 3

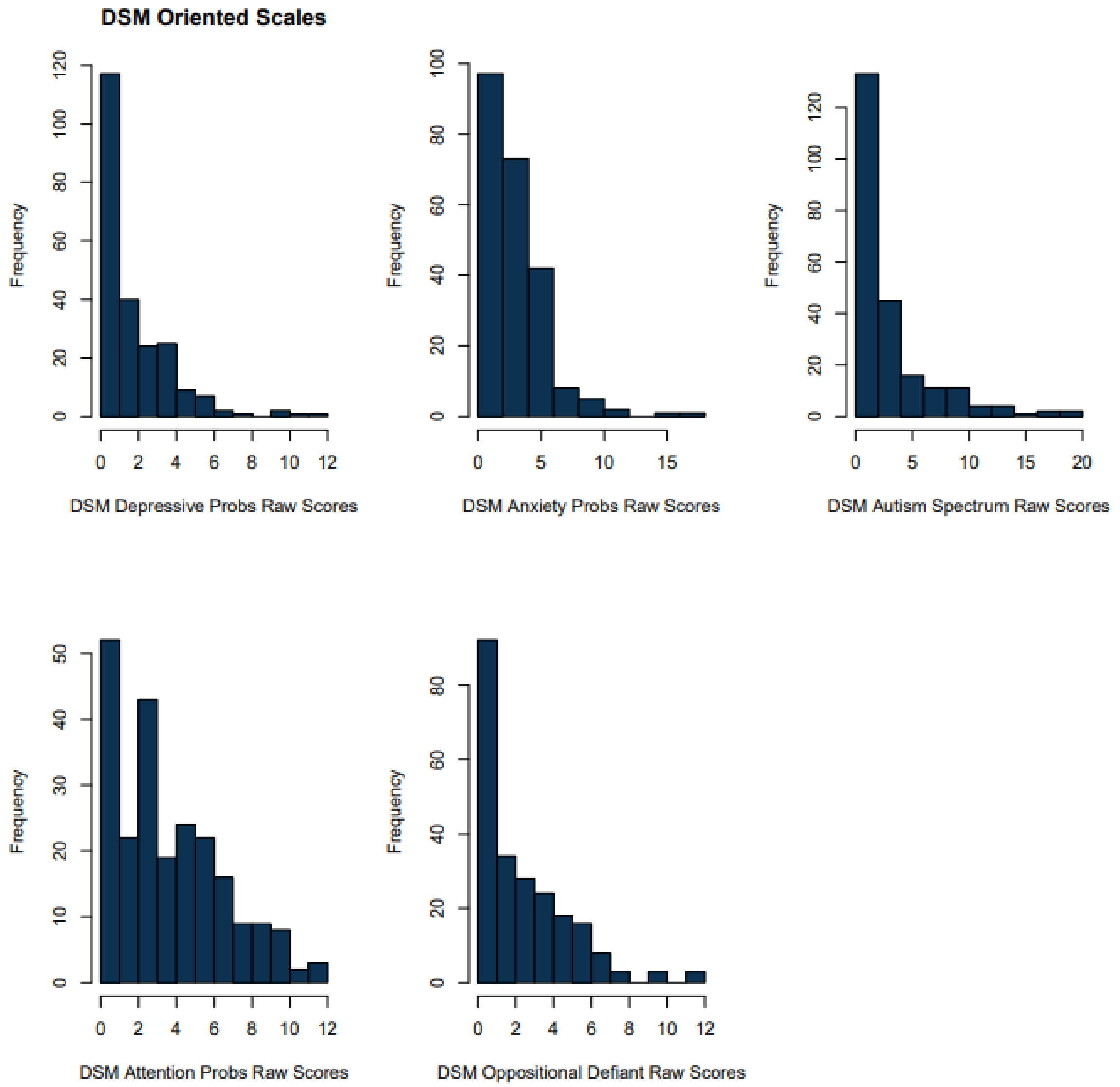

n=229; navy blue histogram bars depict the frequency of raw scores among participants per CBCL DSM-oriented scale.

© 2024 Malin AJ et al. *JAMA Network Open.*

eTable2. CBCL Scores at Age 36-Months in the MADRES Cohort

CBCL Composite or Scale	Arithmetic Mean	Standard Deviation	Range
**Composite *T*-Score**			
Total Problems	47.69	11.60	65
Internalizing	47.13	11.62	61
Externalizing	46.48	10.68	61
**Raw Score**			
Emotionally Reactive	1.75	2.21	14
Anxious-Depressed	2.43	2.29	14
Somatic Complaints	1.46	2.04	14
Withdrawn	1.85	2.62	14
Sleep Problems	2.72	2.58	13
Attention Problems	2.25	2.0	10
Aggressive Behavior	8.03	6.60	38
DSM ADHD Problems	4.08	2.93	12
DSM Depressive Problems	1.98	2.13	12
DSM Anxiety Problems	3.23	2.65	18
DSM Oppositional Defiant Problems	2.72	2.54	12
DSM Autism Spectrum Problems	3.17	3.83	20

Note. n=229.

eTable 3. Poisson Regression Estimating the Risk Ratio for Third Trimester MUFsg in Relation to CBCL Clinical Index Scores

	Clinical Index Variable Scores		
**CBCL Composite Scale**	**RR**	**95%CI**	***p***
Externalizing	0.99	0.89, 1.90	0.18
Internalizing	1.03	1.01, 1.74	**0.04***
Total Problems	1.24	1.18, 1.91	**0.001***

Note. N=229; MUFsg=specific gravity adjusted maternal urinary fluoride; Normal=0; Borderline/Clinical=1; adjusted for maternal age, pre-pregnancy BMI, ethnicity by nativity, maternal education, household income, maternal cohabitation, and child sex; RRs are presented according to an IQR (i.e., 0.68 mg/L) increase in MUFsg. Higher CBCL scores are indicative of more symptoms of neurobehavioral problems.

eTable 4. Sensitivity Analysis Including "Borderline Clinical" With "Non-clinical" Group as the Reference in Logistic Regression of Trimester 3 MUFsg With CBCL Clinical Index Scores

CBCL Index	OR	95%CI	*p*
Externalizing	2.02	0.82, 5.02	0.13
Internalizing	2.05	1.17, 3.59	**0.01***
Total Problems	1.84	1.13, 3.00	**0.02***

Note. n=229; MUFsg=specific gravity adjusted maternal urinary fluoride; Normal/ Borderline=0, Clinical=1; ORs are presented according to an IQR (i.e., 0.68 mg/L) increase in MUFsg. Adjusted for maternal age, pre-pregnancy BMI, ethnicity by nativity, maternal education, household income, maternal cohabitation, and child sex. Higher CBCL scores are indicative of more symptoms of neurobehavioral problems.

© 2024 Malin AJ et al. *JAMA Network Open.*

eTable 5. Sensitivity Analysis of Associations of MUFsg in Trimester 1 With CBCL Clinical Index Scores

CBCL Index	OR	95%CI	*p*
Externalizing	1.99	1.04, 3.80	**0.04***
Internalizing	1.36	0.74, 2.51	0.32
Total Problems	1.71	0.94, 3.09	0.08

Note. n=155; MUFsg=specific gravity adjusted maternal urinary fluoride; Normal=0, Borderline/Clinical=1; ORs are presented according to an IQR (i.e., 0.55 mg/L) increase in MUFsg; adjusted for maternal age, pre-pregnancy BMI, ethnicity by nativity, maternal education, household income, maternal cohabitation, and child sex. Higher CBCL scores are indicative of more symptoms of neurobehavioral problems.

eTable 6. Sensitivity Analysis of Associations of Trimester 1 MUFsg With Composite *T*-Scores, and Syndrome or DSM Scale Raw CBCL Scores at Age 3

CBCL Index or Scale	B	95%CI	*p*
Total Problems [a]	1.55	-1.03, 4.13	0.24
Internalizing [a]	2.41	-0.14, 4.97	0.05
Externalizing [a]	0.95	-1.40, 3.30	0.42
Emotionally Reactive	0.13	-0.03, 0.28	0.11
Anxious-Depressed	0.05	-0.10, 0.20	0.50
Somatic Complaints	0.10	-0.04, 0.25	0.16
Withdrawn	0.12	-0.04, 0.28	0.14
Sleep Problems	0.10	-0.05, 0.26	0.19
Attention Problems [a]	0.28	-0.17, 0.72	0.22
Aggressive Behavior	0.04	-0.14, 0.22	0.68
DSM ADHD Problems [a]	0.50	-0.14, 1.15	0.13
DSM Depressive Problems	0.07	-0.08, 0.21	0.39
DSM Anxiety Problems	0.05	-0.09, 0.20	0.47
DSM Oppositional Defiant Problems [a]	0.25	-0.30, 0.79	0.37
DSM Autism Spectrum Problems	0.16	-0.01, 0.34	0.07

Note. n=155; MUFsg=specific gravity adjusted maternal urinary fluoride; adjusted for maternal age, pre-pregnancy BMI, ethnicity by nativity, maternal education, household income, maternal cohabitation, and child sex; [a] not LN-transformed; B coefficients are presented according to an IQR (i.e., 0.55 mg/L) increase in MUFsg. Higher CBCL scores are indicative of more symptoms of neurobehavioral problems.

© 2024 Malin AJ et al. *JAMA Network Open.*

eTable 7. Associations of MUFsg in Trimester 1 With CBCL Scores Adjusting for Blood Lead

CBCL Index or Scale	B	95%CI	*p*
Total Problems	3.91	0.54, 7.29	**0.02***
Internalizing	4.40	1.09, 7.72	**0.01***
Externalizing	2.82	-0.32, 5.95	0.08
Emotionally Reactive	0.21	0.003, 0.41	**0.05***
Anxious-Depressed	0.11	-0.10, 0.31	0.30
Somatic Complaints	0.22	0.03, 0.42	**0.02***
Withdrawn	0.21	-0.002, 0.43	0.05
Sleep Problems	0.22	0.006, 0.43	**0.04***
Attention Problems [a]	0.60	-0.03, 1.22	0.06
Aggressive Behavior [a]	1.85	-0.08, 3.78	0.06
DSM ADHD Problems [a]	1.03	0.16, 1.90	**0.02***
DSM Depressive Problems	0.14	-0.06, 0.35	0.18
DSM Anxiety Problems	0.14	-0.05, 0.34	0.15
DSM Oppositional Defiant [a] Problems	0.63	-0.06, 1.32	0.07
DSM Autism Spectrum Problems	0.24	-0.004, 0.48	0.05

Note. n=121; MUFsg=specific gravity adjusted maternal urinary fluoride; adjusted for maternal age, pre-pregnancy BMI, ethnicity by nativity, maternal education, household income, maternal cohabitation, trimester 1 blood lead and child sex; [a] not LN-transformed; B coefficients are presented according to an IQR (i.e., 0.55 mg/L) increase in MUFsg. Higher CBCL scores are indicative of more symptoms of neurobehavioral problems. P for Emotionally Reactive < .05 but equals 0.05 due to rounding.

eTable 8. Associations of MUFsg in Trimester 3 With CBCL Scores Adjusting for Trimester 1 Blood Lead

CBCL Index or Scale	B	95%CI	*p*
Total Problems [a]	2.84	0.37, 5.31	**0.03***
Internalizing [a]	3.16	0.69, 5.62	**0.01***
Externalizing [a]	1.54	-0.73, 3.80	0.18
Emotionally Reactive	0.18	0.03, 0.32	**0.02***
Anxious-Depressed	0.12	-0.03, 0.27	0.10
Somatic Complaints	0.21	0.07, 0.36	**0.004***
Withdrawn	0.16	0.002, 0.32	**0.05***
Sleep Problems	0.08	-0.07, 0.24	0.30
Attention Problems [a]	0.16	-0.30, 0.62	0.49
Aggressive Behavior	0.11	-0.06, 0.28	0.19
DSM ADHD Problems [a]	0.32	-0.32, 0.96	0.33
DSM Depressive Problems	0.14	-0.01, 0.28	0.07
DSM Anxiety Problems	0.13	-0.01, 0.27	0.08
DSM Oppositional Defiant Problems	0.07	-0.08, 0.22	0.33
DSM Autism Spectrum Problems	0.21	0.03, 0.39	**0.02***

Note. n=123; MUFsg=specific gravity adjusted maternal urinary fluoride; adjusted for maternal age, pre-pregnancy BMI, ethnicity by nativity, maternal education, household income, trimester 1 blood lead, maternal cohabitation, and child sex; [a] not LN-transformed; B coefficients are presented according to an IQR (i.e., 0.68 mg/L) increase in MUFsg. Higher CBCL scores are indicative of more symptoms of neurobehavioral problems. P for Withdrawn < .05 but equals 0.05 due to rounding.

© 2024 Malin AJ et al. *JAMA Network Open.*

<u>eTable 9.</u> Associations of MUFsg in Trimester 3 With CBCL Clinical Index Scores Adjusting for Trimester 1 Blood Lead

CBCL Index	OR	95%CI	*p*
Externalizing	1.30	0.69, 2.44	0.41
Internalizing	2.62	1.26, 5.44	**0.01***
Total Problems	2.92	1.36, 6.26	**0.006***

Note. n=123; MUFsg=specific gravity adjusted maternal urinary fluoride; Normal=0, Borderline/Clinical=1; ORs are presented according to an IQR (i.e., 0.68 mg/L) increase in MUFsg; adjusted for maternal age pre-pregnancy BMI, ethnicity by nativity, maternal education, household income, maternal cohabitation, trimester 1 blood lead and child sex. Higher CBCL scores are indicative of more symptoms of neurobehavioral problems.

<u>eTable 10</u>. Associations of MUFsg in Trimester 3 With CBCL Scores Among Women Who Fasted for >= 8 Hours

CBCL Index or Scale	B	95% CI	*p*
Total Problems [a]	2.25	0.28, 4.21	**0.03***
Internalizing [a]	2.39	0.45, 4.33	**0.02***
Externalizing [a]	1.50	-0.31, 3.30	0.10
Emotionally Reactive	0.13	0.01, 0.25	**0.03***
Anxious-Depressed	0.12	-0.04, 0.27	0.15
Somatic Complaints	0.15	0.04, 0.27	**0.008***
Withdrawn	0.11	-0.02, 0.23	0.10
Sleep Problems	0.06	-0.07, 0.18	0.36
Attention Problems [a]	0.25	-0.10, 0.61	0.17
Aggressive Behavior	0.11	-0.04, 0.25	0.16
DSM ADHD Problems [a]	0.43	-0.09, 0.95	0.10
DSM Depressive Problems	0.10	-0.01, 0.22	0.08
DSM Anxiety Problems	0.11	-0.005, 0.22	0.06
DSM Oppositional Defiant Problems	0.07	-0.06, 0.19	0.29
DSM Autism Spectrum Problems	0.18	0.04, 0.32	**0.01***

Note. n=192; MUFsg=specific gravity adjusted maternal urinary fluoride; [a]not LN transformed; adjusted for maternal age, pre-pregnancy BMI, ethnicity by nativity, maternal education, household income, maternal cohabitation, and child sex; Beta coefficients are presented according to an IQR (i.e., 0.68 mg/L) increase in MUFsg; n=4 had missing data for fasting duration status. Higher CBCL scores are indicative of more symptoms of neurobehavioral problems.

<u>eTable 11.</u> Trimester 3 MUFsg in Relation to CBCL Clinical Index Scores Among Women Who Fasted for >= 8 Hours

CBCL Index	OR	95%CI	*p*
Externalizing	1.38	0.85, 2.26	0.20
Internalizing	1.50	0.94, 2.39	0.09
Total Problems	1.84	1.16, 2.94	**0.01***

Note. N=192; MUFsg=specific gravity adjusted maternal urinary fluoride; Normal=0; Borderline/Clinical=1; adjusted for maternal age, pre-pregnancy BMI, ethnicity by nativity, maternal education, household income, maternal cohabitation, and child sex; ORs are presented according to an IQR (i.e., 0.68 mg/L) increase in MUFsg; n=4 had missing data for fasting duration status. Higher CBCL scores are indicative of more symptoms of neurobehavioral problems.

© 2024 Malin AJ et al. *JAMA Network Open.*

eTable 12. Associations of MUFsg in Trimester 3 With CBCL Scores Including Women Who Smoked During Pregnancy

CBCL Index or Scale	B	95% CI	*p*
Total Problems [a]	2.04	0.21, 3.86	**0.03***
Internalizing [a]	2.20	0.41, 4.01	**0.02***
Externalizing [a]	1.37	-0.34, 3.08	0.12
Emotionally Reactive	0.12	0.01, 0.23	**0.03***
Anxious-Depressed	0.08	-0.03, 0.18	0.16
Somatic Complaints	0.18	0.07, 0.28	**0.001***
Withdrawn	0.08	-0.03, 0.20	0.17
Sleep Problems	0.06	-0.05, 0.17	0.28
Attention Problems [a]	0.21	-0.11, 0.53	0.20
Aggressive Behavior	0.10	-0.04, 0.238	0.15
DSM ADHD Problems [a]	0.40	-0.07, 0.87	0.10
DSM Depressive Problems	0.09	-0.02, 0.19	0.11
DSM Anxiety Problems	0.11	0.001, 0.21	**0.05***
DSM Oppositional Defiant Problems	0.07	-0.04, 0.19	0.23
DSM Autism Spectrum Problems	0.16	0.04, 0.29	**0.01***

Note. n=235; MUFsg=specific gravity adjusted maternal urinary fluoride; [a]not LN transformed; adjusted for maternal age, pre-pregnancy BMI, ethnicity by nativity, maternal education, household income, maternal cohabitation, child sex and maternal smoking status during pregnancy; Beta coefficients are presented according to an IQR (i.e., 0.68 mg/L) increase in MUFsg. Higher CBCL scores are indicative of more symptoms of neurobehavioral problems. P for DSM Anxiety < .05 but equals 0.05 due to rounding.

eTable 13. Trimester 3 MUFsg in Relation to CBCL Clinical Index Scores Including Women Who Smoked During Pregnancy

CBCL Index	OR	95%CI	*p*
Externalizing	1.22	0.77, 1.95	0.40
Internalizing	1.43	0.93, 2.20	0.10
Total Problems	1.74	1.13, 2.68	**0.01***

Note. N=235; MUFsg=specific gravity adjusted maternal urinary fluoride; Normal=0; Borderline/Clinical=1; adjusted for maternal age, pre-pregnancy BMI, ethnicity by nativity, maternal education, household income, maternal cohabitation, child sex and maternal smoking status during pregnancy; ORs are presented according to an IQR (i.e., 0.68 mg/L) increase in MUFsg. Higher CBCL scores are indicative of more symptoms of neurobehavioral problems.

© 2024 Malin AJ et al. *JAMA Network Open.*

eTable 14. Associations of Average MUFsg Across Trimesters 1 and 3 With CBCL Scores

CBCL Index or Scale	B	95% CI	*p*
Total Problems [a]	2.85	0.02, 5.77	**0.05***
Internalizing [a]	3.98	1.19, 6.77	**0.006***
Externalizing [a]	1.44	-1.14, 4.02	0.27
Emotionally Reactive	0.20	0.03, 0.37	**0.02***
Anxious-Depressed	0.16	-0.003, 0.33	0.06
Somatic Complaints	0.22	0.06, 0.38	**0.008***
Withdrawn	0.19	0.008, 0.36	0.04
Sleep Problems	0.10	-0.07, 0.27	0.25
Attention Problems [a]	0.23	-0.27, 0.72	0.37
Aggressive Behavior	0.10	-0.09, 0.30	0.30
DSM ADHD Problems [a]	0.45	-0.26, 1.17	0.21
DSM Depressive Problems	0.14	-0.02, 0.30	0.09
DSM Anxiety Problems	0.16	0.004, 0.32	**0.05***
DSM Oppositional Defiant Problems	0.07	-0.10, 0.24	0.40
DSM Autism Spectrum Problems	0.27	0.07, 0.46	**0.007***

Note. n=154; MUFsg=specific gravity adjusted maternal urinary fluoride; [a]not LN transformed; adjusted for maternal age, pre-pregnancy BMI, ethnicity by nativity, maternal education, household income, maternal cohabitation, and child sex; Beta coefficients are presented according to an IQR (i.e., 0.59 mg/L) increase in MUFsg. Higher CBCL scores are indicative of more symptoms of neurobehavioral problems. P for Total Problems and DSM Anxiety < .05 but equal 0.05 due to rounding.

eTable 15. Average MUFsg Across Trimesters 1 and 3 in Relation to CBCL Clinical Index Scores

CBCL Index	OR	95%CI	*p*
Externalizing	1.64	0.78, 3.42	1.91
Internalizing	2.28	1.16, 4.50	**0.02***
Total Problems	2.43	1.23, 4.84	**0.01***

Note. N=154; MUFsg=specific gravity adjusted maternal urinary fluoride; Normal=0; Borderline/Clinical=1; adjusted for maternal age, pre-pregnancy BMI, ethnicity by nativity, maternal education, household income, maternal cohabitation, and child sex; ORs are presented according to an IQR (i.e., 0.59 mg/L) increase in MUFsg. Higher CBCL scores are indicative of more symptoms of neurobehavioral problems.

© 2024 Malin AJ et al. *JAMA Network Open.*
