# Supplementary material for: Maternal Urinary Fluoride and Child Neurobehavior at Age 36 Months
Source: JAMA Netw Open. 2024 May 20;7(5):e2411987. doi: 10.1001/jamanetworkopen.2024.11987 (PMC11107298; doi:10.1001/jamanetworkopen.2024.11987)
Supplement: Supplement 2. — Data Sharing Statement [file jamanetwopen-e2411987-s002.pdf]

Data Sharing Statement

Malin. Maternal Urinary Fluoride and Child Neurobehavior at Age 36-Months. *JAMA Netw Open*. Published May 20, 2024. doi:10.1001/jamanetworkopen.2024.11987

Data
Data available: Yes
Data types: Deidentified participant data, Data dictionary
How to access data: Deidentified data will be made available upon reasonable request with review and approval from our data sharing committee.
When available: With publication

Supporting Documents
Document types: None

Additional Information
Who can access the data: Deidentified data will be made available upon reasonable request
Types of analyses: Deidentified data will be made available upon reasonable request with review and approval from our data sharing committee.
Mechanisms of data availability: Deidentified data will be made available upon reasonable request with review and approval from our data sharing committee, and with a signed data access agreement.
